# Sexual Health in Women with Inflammatory Bowel Diseases: A Narrative Review

**DOI:** 10.3390/healthcare13070716

**Published:** 2025-03-24

**Authors:** Caterina Mercuri, Vincenzo Bosco, Raúl Juárez-Vela, Assunta Guillari, Silvio Simeone, Patrizia Doldo

**Affiliations:** 1 Department of Clinical and Experimental Medicine, University of Catanzaro Magna Graecia, 88100 Catanzaro, Italy; silvio.simeone@unicz.it (S.S.); doldo@unicz.it (P.D.); 2 Department of Medical and Surgical Sciences, University Hospital Mater Domini, Magna Graecia University, 88100 Catanzaro, Italy; vincenzo.bosco@unicz.it; 3 Faculty of Health Sciences, University of La Rioja, 26006 Logrono, Spain; raul.juarez@unirioja.es; 4 Department of Translational Medical Science, University of Naples Federico II, 80131 Naples, Italy; assunta.guillari@unina.it

**Keywords:** inflammatory bowel diseases (IBD), sexual health, women’s experiences, body image, quality of life

## Abstract

**Background/Objectives:** Inflammatory bowel diseases (IBDs), such as Crohn’s disease and ulcerative colitis, have a significant impact on overall well-being. Sexual health, a critical component of overall well-being, is often compromised in individuals with IBD, especially in women, owing to physical, psychological, and social factors. This narrative review aims to synthesize the fragmented existing evidence on the impact of IBD on women’s sexual health by examining clinical manifestations along with patients’ perceptions and lived experiences. **Methods:** Five databases (CINAHL Complete, Medline, APA PsycInfo, APA PsycArticles, and Psychology and Behavioral Sciences Collection) were searched using keywords related to IBD, sexual health, and women’s experiences. Fifteen studies that met the predefined inclusion and exclusion criteria were analyzed. **Results:** Women with IBD often perceive their bodies as “damaged” or “mutilated,” which profoundly affects their self-esteem and sexual satisfaction. Physically, debilitating symptoms such as abdominal pain, diarrhea, and chronic fatigue limit sexual desire and intimacy. Psychological factors, including anxiety, depression, and negative body image, exacerbate these challenges, and compromise emotional well-being and intimate relationships. Social stigma further isolates patients, making it more difficult for them to communicate their sexual needs to both partners and healthcare providers. Strategies such as psychological support, health education, and promoting open communication with partners emerge as promising avenues to improve sexual health and quality of life. **Conclusions:** IBD profoundly affects women’s sexual health by intertwining physical, psychological, and social challenges. A holistic and personalized clinical approach that incorporates sexual well-being into routine care is essential to improve patients’ quality of life.

## 1. Introduction

Inflammatory bowel diseases (IBD), including Crohn’s disease and ulcerative colitis, are chronic conditions with an unpredictable course, marked by flare-ups and remission phases [1,2]. These disorders mainly affect young adults and require lifelong management [3]. These disorders involve debilitating symptoms such as abdominal pain, diarrhea, weight loss, bloating, and fatigue [4]. Additionally, they are frequently associate with extra-intestinal manifestations affecting up to 50% of patients [5]. These manifestations can involve several body systems, including musculoskeletal, ocular, skin, and hepatobiliary [6].

In addition to physical effects, IBD can lead a numerous emotional and relational consequences [7,8,9]. Patients frequently experience diminished self-esteem, anxiety, and depression [10,11,12], which further exacerbate their overall quality of life [13,14,15]. The unpredictability of symptoms, along with the social stigma associated with bowel urgency and incontinence, restricts social interactions and work performance [16,17,18,19,20].

This situation intensifies psychological distress and contributes to sexual problems and dissatisfaction [21,22].

Sexual health has a significant impact on an individual’s overall quality of life [23]. The World Health Organization defines sexual health as a state of physical, emotional, mental, and social well-being in relation to sexuality [24]. IBD, frequently diagnosed between the ages of 20 and 40 [25], coincides with a crucial period for the development of body image, intimate relationships, and family planning [26,27,28,29,30]. Given that sexuality is a primary determinant of quality of life, particularly among younger patients, issues related to sexual function and intimacy are among the most pressing concerns for individuals with IBD [31,32,33]. A series of studies conducted by Timmer et al. [34,35] provided substantial evidence of the detrimental impact of IBD on sexual health, underscoring how this condition significantly affects patients’ overall well-being. Similarly, Mantzouranis et al. [36] confirmed a significant impairment of sexual health among patients with IBD.

Women with IBD are especially susceptible to negative sexual health outcomes, which can undermine essential aspects of their emotional and relational well-being [37]. These adverse arise from a combination of physiological, hormonal, relational, and sociocultural factors [38,39,40,41].

Knowles et al. [42] reported that a quarter of women perceive IBD as a significant barrier to intimate relationships. Studies have estimated that up to 53% of women with IBD experience a lower quality of sexual health compared to the general population, with depression being identified as an independent predictor of this impairment [43]. Physically, symptoms such as pelvic pain, chronic fatigue, and the side effects of drug therapies diminish sexual desire and hinder intimacy [21,44,45]. This is further exacerbated by anxiety related to disease management, which intensifies negative body perceptions and diminishes the sexual quality of life [21,31,42].

Although sexual health is a crucial aspect of overall well-being, the understanding of sexual health in women with IBD remains limited and fragmented. Through a careful analysis of the literature, the aim of this narrative review is to synthesize studies that have investigated how IBD affects sexual health in women.

This type of narrative review not only provides an overview of current knowledge, but also identifies gaps and opportunities for research [46].

Enhanced understanding could foster greater awareness of this issue among health professionals and patients, thereby supporting the development of an effective and efficient care approach and ultimately improving overall health and well-being.

## 2. Materials and Methods

### 2.1. Research Strategies

In this literature review, a narrative analysis was conducted by analyzing studies of various designs [47].

A narrative review was conducted because it is a basic methodological tool for synthesizing a wide range of literature on a complex topic, providing a critical and integrative perspective on the available evidence.

The research was conducted on five main databases: CINAHL Complete, Medline, APA PsycInfo, APA PsycArticles, and the Psychology and Behavioral Sciences Collection.

A search string consisting of the following keyword combinations was adopted:

- “Sexual health” AND (“IBD” OR “inflammatory bowel disease”) AND (“women” OR “female” OR “females”) AND (“lived experiences” OR “perceptions” OR “attitudes” OR “views” OR “phenomenology”).

The string was optimized to ensure the identification of relevant articles, considering both specific terms and synonyms commonly used in the literature.

The research was carried out between October 2024 and January 2025.

### 2.2. Inclusion and Exclusion Criteria

The articles selected for inclusion had to meet the following criteria:

Inclusion Criteria
  (a) Studies published in the English language;
  (b) Studies conducted in adult subjects;
  (c) Peer-reviewed articles of any study design, including systematic reviews, observational studies, and intervention studies;
  (d) Articles related to sexual health in women with IBD (or clearly extrapolatable).
Exclusion Criteria
  (a) Studies published not in the English language;
  (b) Studies conducted in pediatric populations;
  (c) Letters to the editor and conference abstracts;
  (d) Articles unrelated to sexual health in women with IBD.

### 2.3. Selection Process

The search strategy produced a total of 355 results, distributed as follows:

- 179 items from CINAHL Complete;
- 60 from Medline;
- 1 from APA PsycInfo;
- 9 by APA PsycArticles;
- 106 from Psychology and Behavioral Sciences Collection.

After the removal of 14 duplicates, 341 articles were screened for titles and abstracts. Of these, 294 articles were excluded as not relevant to the objectives of the study. Of the remaining 47 articles, 46 were screened in full, because 1 full article was not found. At the end of the process, 15 articles were included, while 31 were excluded as they did not meet the inclusion criteria.

**Figure 1:**
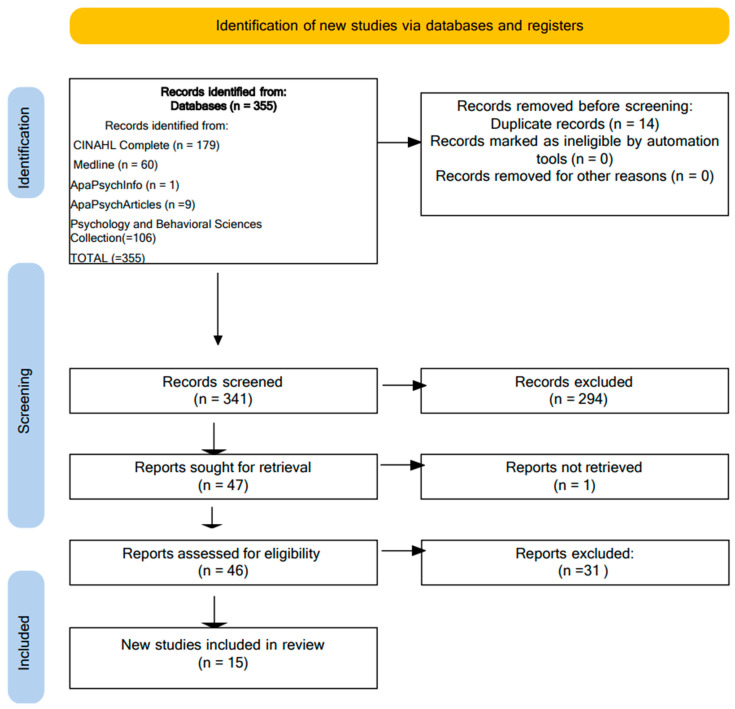
PRISMA flow diagram [48].

Two reviewers conducted the selection independently, with the supervision of two experts to resolve any discrepancies. The authors achieved a 100% agreement on the inclusion and exclusion of articles after a discussion in which individual articles were evaluated according to the inclusion criteria. The detailed process of article selection is shown in Figure 1 based on the PRISMA (Preferred Reporting Items for Systematic Reviews and Meta-Analysis) model [48].

### 2.4. Study Selection and Categorization Process

**Figure 2:**
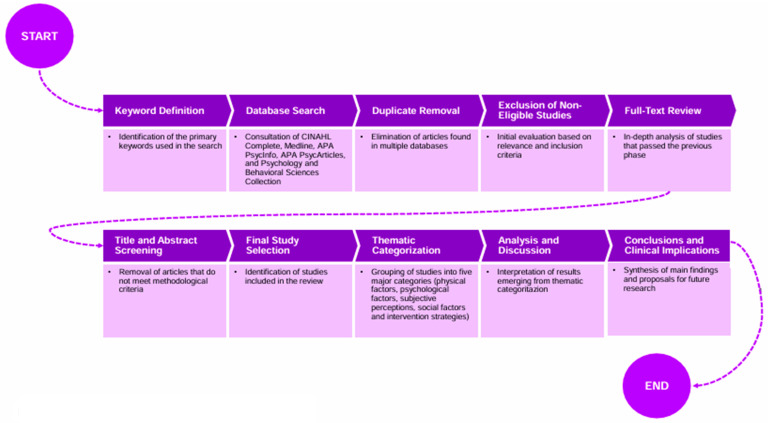
Block diagram of the literature selection process.

A block diagram illustrating the process of study selection and categorization is illustrated in Figure 2 to provide greater methodological clarity. This visual representation outlines the stages of identification, screening, and inclusion of the articles analyzed, highlighting the criteria applied at each step.

### 2.5. Quality Appraisal

The evaluation of the methodological quality of the included studies was conducted through the utilization of the QuADS tool (Quality Assessment with Diverse Studies). This tool, selected for its reliability and validity across various study designs such as quantitative, qualitative, mixed, and multimethod studies [49,50]. To ensure rigorous evaluation, two independent reviewers conducted the quality assessment, resolving any scoring discrepancies through discussion. The final score for each study was determined as a percentage, calculated by the ratio of the total score to the total criteria score [final score = total score of each study/total criteria score × 100%] [49].

A summary of the quality appraisal results is presented in Table 1.

## 3. Results

The data of the selected studies were grouped into thematic categories to facilitate an in-depth understanding of the research objective. The main characteristics of the studies are summarized in Table 2.

The review demonstrated multiple dimensions of how IBD affects women’s sexual health.

The results of the review highlight how inflammatory bowel disease (IBD) compromises women’s sexual health through a combination of debilitating physical symptoms and a significant psychological burden, which affect sexual desire, satisfaction, and the quality of intimate relationships. Additionally, beyond the physiological aspects related to the disease and its treatments, profound emotional and social implications arise, contributing to a complex and multidimensional experience of sexuality.

The impairment of sexual health is not solely dependent on disease activity but is also influenced by patients’ perceptions of their bodies and the quality of their interactions with their partners and social environment. In particular, body image and emotional support play a key role in modulating the impact of the disease on sexuality, affecting the psychological and relational well-being of women with IBD.

The results are grouped into five main thematic categories.

### 3.1. Physical Factors

- Symptoms of active disease: Physical symptoms such as abdominal pain, diarrhea, bloating, and chronic fatigue represent a significant barrier to sexuality. Domislovic et al. [52] reported that 75% of women with IBD experience a reduction in the quality of sexual intercourse during the active phases of the disease. Zhang et al. [63] estimated an overall prevalence of sexual dysfunction (SD) of 53%, increasing to 65% in the presence of severe symptoms. White et al. [62] showed that over 40% of patients avoid intimate situations due to embarrassing bowel symptoms such as leakage or urgency.
- Surgeries: Surgeries, particularly the creation of ostomies, have a significant impact on sexual health. Ghazi et al. [55] showed that 40–66% of surgery patients experience sexual dysfunction, attributing this condition to the physical changes and emotional impact of the operations. McIntosh et al. [58] observed that 58% of ostomy patients report sexual difficulties, often associated with insecurities related to the management of the ostomy itself.

### 3.2. Psychological Factors

- Anxiety and Depression: Boyd et al. [51] report that depression is a particularly strong predictor of sexual dysfunction, with IBD patients experiencing depression reporting difficulties in reaching orgasm, reduced sexual desire, satisfaction, and frequency of intercourse. Zhang et al. [63] identified depression (OR 6.14, 95% CI 2.81–13.43) and anxiety (OR 3.21, 95% CI 1.69–6.09) as significant risk factors for a decline in sexual quality of life. Their analysis indicates that women with moderate-to-severe depressive symptoms are more than six times more likely to experience intimacy-related difficulties compared to those without depression. Zhang et al. [63] further confirm that disease perception significantly impacts mental health, influencing anxiety, depression, and family dynamics, which in turn directly affect sexual function and intimacy satisfaction. Additionally, patients with active disease report significantly higher rates of severe depression compared to those in remission, along with lower social support, poorer disease-specific quality of life, and reduced overall well-being. Ghazi et al. [55] highlight that increased disease activity and the presence of depression are key predictors of reduced sexual function. Their study also found that 72% of patients with anxiety and 63% with depression report significant sexual difficulties, reinforcing the link between mental health and sexual function. Moreover, patients with higher anxiety scores report lower levels of sexual desire and satisfaction, regardless of disease activity. These findings highlight a bidirectional link between IBD and mental health: on one hand, the disease itself and its debilitating symptoms contribute to increased levels of anxiety and depression; on the other, psychological distress exacerbates sexual difficulties, increasing the risk of emotional isolation and impairing relationship quality, as emphasized by Boyd et al. [51].
- Negative body perception: Boyd et al. [51] and Fourie et al. [53] reported that more than 60 per cent of women with IBD experience an altered body perception, influenced by scarring, bloating, and loss of bowel control. This negative perception reduces self-esteem and increases the risk of emotional isolation, further aggravating IBD. Negative body perception is closely related to the fear of being judged by a partner, as noted by Knowles et al. [42]. McIntosh et al. [58] highlighted that negative body perception is particularly pronounced in patients with surgical scarring, making it more difficult to return to a satisfying sexual life. Smith et al. [61] found that women with ileostomies struggle with body image and intimate relationships, although appropriate support can facilitate personal growth and help them to overcome these challenges.

### 3.3. Women’s Perceptions and Experiences

- Subjective experience: Women with IBD often describe their bodies as ’mutilated’ or ’damaged’, which has a significant impact on their self-esteem and intimate relationships [51,53]. Pires et al. [60] highlighted that 48% of women feel inadequate in relation to their partner’s expectations, contributing to a sense of emotional isolation. According to Knowles et al. [42], 25% of women perceive IBD as a significant obstacle to maintaining intimate relationships.
- Relationships and support: Relationships characterized by empathy and support improve sexual quality of life. However, a lack of mutual communication, reported in 34% of couples [60], increases emotional and physical distance. This highlights the need for interventions that promote dialogue between partners. Fretz et al. [54] found that many patients avoid discussing their sexual difficulties for fear of misunderstanding or negative judgement. White et al. [62] found that improved dialogue and mutual support in the couple can alleviate difficulties related to sexuality and improve relationship well-being.
- Clinical Invisibility: Boyd et al. [51] reported that only 14% of gastroenterologists regularly address sexual health during clinical visits. White et al. [62] reported that a significant proportion of patients expressed a desire for healthcare professionals to address the impact of IBD on sexual health, emphasizing the importance of integrating this topic into clinical discussions.

### 3.4. Social and Relational Factors

- Emotional isolation: Igerc et al. [56] observed that 58% of women with IBD feel isolated due to the disease, negatively affecting both their intimate and social relationships. Fourie et al. [53] pointed out that insufficient social support exacerbates emotional distress, contributing to the deterioration of personal relationships. Ellul et al. [27] highlighted that adequate social support serves as a crucial protective factor in enhancing sexual quality of life.
- Social impact: The stigma associated with IBD symptoms, such as leakage and bowel urgency, limits social interactions and affects quality of life. Boyd et al. [51] and Fourie et al. [53] reported that more than 50 per cent of patients feel stigmatized, with a significant impact on emotional and relational well-being.

### 3.5. Solutions and Strategies

- Multidisciplinary approaches: Interventions combining psychological support, education, and physical rehabilitation have proven to be particularly effective. Ellul et al. [27] reported that 68% of patients treated with multidisciplinary programs experienced a significant improvement in the quality of their sexual life. Pelvic floor rehabilitation produced positive results in 72% of cases [56]. Nisihara et al. [59] emphasized the significance of a multidisciplinary approach tailored to the specific needs of patients, which plays a key role in enhancing their overall quality of life. Kanar et al. [57] highlighted that the treatment of patients with IBD should be individualized, taking into account the aggressiveness of the disease, the goals of treatment, and the tolerability of various medications. These findings underline the importance of personalized and integrative approaches in improving sexual health and overall well-being in patients with IBD.
- Standardized instruments: The use of assessment tools such as the Female Sexual Function Index (FSFI) are instrumental in the early detection of sexual dysfunction problems early on. Zhang et al. [63] found that 39% of patients present with DS according to the FSFI, allowing for targeted interventions. Furthermore, the Sexual Quality of Life Questionnaire (SQOL-F) improved sexual satisfaction in 56% of patients undergoing targeted interventions [62]. The integration of such tools into clinical protocols facilitates individualized management, addressing the specific issues of patients with IBD.

**Table 2:** Data extraction of included articles.

Author, Year, Country	Objective	Method	Sample	Main Findings	Strengths	Limitations	Pros—Cons
Boyd, 2022, USA [51]	Overview of sexual dysfunction in female IBD patients	Review study	N/A	Sexual dysfunction higher in IBD, linked to disease type and mental health	Comprehensive review on sexual dysfunction in IBD	Lack of uniformity in cited methodologies	Pros: Broad review; Cons: Data inconsistency across studies
Domislovic, 2021, Croatia [52]	Assessing prevalence of sexual dysfunction	Cross-sectional study	202 patients (122 men, 80 women)	Sexual dysfunction prevalence: 18% men, 75% women	High response rate (85.5%)	No control group for comparison	Pros: High response rate; Cons: No control group
Ellul, 2016, Mediterranean countries [27]	Perceptions of reproductive health in women with IBD	Multicenter prospective study	348 women with IBD	Misconceptions about fertility and pregnancy in IBD	Large Multicenter sample	Recall bias possible	Pros: Large and diverse sample; Cons: Recall bias
Fourie, 2018, UK, Ireland, USA, South Africa [53]	Experiences of IBD patients discussing sexual health with HCPs	Phenomenological qualitative study	43 participants (32 women, 11 men)	Sexual health is rarely addressed in IBD consultations	Includes perspectives of sexual minorities	Small sample limits generalizability	Pros: Unique qualitative insight; Cons: Small sample
Fretz, 2024, Canada, USA, UK [54]	Impact of IBD on sexuality and health experiences	Multimethod study (qualitative and quantitative)	470 participants	Only 5.7% receive adequate support for sexual health	Diverse sample, multimethod approach	Overrepresentation of white, educated women	Pros: Mixed-method approach; Cons: Population bias
Ghazi, 2015, USA [55]	Multifactorial causes of sexual dysfunction in IBD	Clinical literature review	Not applicable	Depression and anxiety major contributors to sexual dysfunction	In-depth discussion on risk factors	Lacks empirical patient data	Pros: Strong theoretical framework; Cons: Lacks primary patient data
Igerc, 2023, Austria [56]	Sexual well-being needs in chronic disease patients	Scoping review (JBI framework)	50 articles included	Patients desire discussions on sexual health	Broad literature scope	No patient-reported outcomes included	Pros: Comprehensive literature review; Cons: No patient outcomes
Kanar, 2017, USA [57]	Impact of biologics on sexual health in IBD	Monocentric cross-sectional study	125 patients (70% Crohn’s, 30% UC)	Biologics affect libido and satisfaction, needing individualized treatment	First study on biologics and sexual health in IBD	Single-center study limits findings	Pros: Novel focus on biologics; Cons: Monocentric design
Knowles, 2018, UK [42]	Effects of body image on intimacy in IBD patients	Survey-based analysis	210 patients	Body image issues strongly linked to intimacy avoidance	First study linking body image and intimacy	Subjective reporting may influence results	Pros: First study on body image and sex; Cons: Self-reported
McIntosh, 2020, Australia [58]	Experiences of ostomy patients on sexual health	Qualitative interviews	35 ostomy patients	Ostomy patients report significant decline in sexual activity	In-depth analysis of ostomy impact on sex life	Small sample size	Pros: First to assess ostomy impact; Cons: Small sample
Nisihara, 2020, Brazil [59]	IBD activity and its impact on sexual dysfunction	Longitudinal cohort study	187 women with IBD	Higher IBD activity = higher sexual dysfunction prevalence	First longitudinal study on IBD and sexual dysfunction	Longitudinal follow-up still limited	Pros: Longitudinal insight; Cons: Limited follow-up
Pires, 2022, Portugal [60]	Quality of life and sexual health in women with IBD	Observational study	154 female IBD patients	Lower sexual QOL in active disease vs. remission	Well-validated QOL tools used	Lack of intervention analysis	Pros: Well-validated tools; Cons: No intervention testing
Smith, 2017, USA [61]	Long-term impact of IBD-related surgeries on sexual health	Qualitative study	21 post-surgical IBD patients	Surgical complications worsen long-term sexual health	Post-surgical impact explored	Post-surgical outcomes vary widely	Pros: Post-surgical insight; Cons: High variability
White, 2021, USA [62]	Patient perspectives on discussing sexual health with providers	Expert review	N/A	Patients prefer structured sexual health discussions	Patient-reported outcomes improve validity	Limited generalizability across healthcare settings	Pros: Structured patient reporting; Cons: Limited setting generalization
Zhang, 2020, China [63]	Mental health, depression, and sexual dysfunction in IBD patients	Systematic review and meta-analysis	N/A	Depression and anxiety significantly correlate with sexual dysfunction in IBD patients	Strong mental health correlations	Self-reported data may include bias	Pros: Strong mental health correlations; Cons: Self-report bias

Legend: N/A: Not Applicable; PROS: Advantages of the study; CONS: Disadvantages of the study.

## 4. Discussion

This narrative review highlights the profound impact of inflammatory bowel disease (IBD) on women’s sexual health, intertwining physical, psychological, and social aspects with patients’ subjective experiences. Sexual health, defined as the capacity to engage in positive sexual experiences and responses, constitutes a vital component of overall well-being, which is frequently compromised in individuals with IBD [64,65].

Similar to other chronic diseases, IBD can negatively sexual function due to a confluence of disease-related symptoms, psychological distress, and treatment side effects. In addition to the direct effects of their condition, patients with chronic diseases may experience diminished self-esteem and altered body-image perceptions, which can affect intimate relationships [66]. Furthermore, certain treatments may induce sexual side effects, such as erectile dysfunction or decreased libido [67]. For example, women with type 1 and type 2 diabetes or hypertension report a lower sexual quality of life compared to women without chronic conditions [68]. Similarly, joint conditions such as rheumatoid arthritis and psoriatic arthritis are associated with elevated rates of sexual dysfunction, affecting pleasure, desire, arousal, and orgasm [69].

Neurological conditions such as stroke and Parkinson’s disease can diminish sexual activity and satisfaction owing to motor and sensory impairments [70]. Similar challenges are evident in patients with IBD, where pharmacological and surgical treatments can negatively affect sexual function. Additionally, systemic symptoms including abdominal pain, fatigue, and bowel dysfunction affect sexual desire and the quality of intimacy, similar to other chronic diseases [31,64].

However, IBD is characterized by distinct features, including involvement of the gastrointestinal tract and potential surgical complications, including perianal fistulas, which may further affect sexual activity [31,36]. One unique aspect is the unpredictable course of this disease, marked by alternating remissions and flare-ups, which significantly affects quality of life and relationship dynamics. Unlike other chronic conditions with more stable symptoms, this variability makes the management of sexual health in IBD particularly complex, with implications for psychological well-being and partner relationships [60,64,71].

The prevalence of sexual health impairment among women with IBD is significantly high. A study conducted by Nisihara et al. [59] reported a prevalence of 82.5%,which was significantly higher than that observed in healthy controls [63].

These findings align with the results of Domislovic et al. [52], who highlighted that the prevalence of sexual dysfunction is generally higher in women with IBD compared to men, with gender differences being particularly pronounced in specific age groups. Specifically, the study indicated that the negative impact on sexual health in men is more prevalent between the ages of 21 and 30, with a subsequent increase after the age of 51. In contrast, women experience sexual dysfunction more consistently throughout the course of the disease [52]. These findings are corroborated by with those of longitudinal study conducted by Shmidt [72], who found that women with IBD who experienced sexual dysfunction did not exhibit significant improvement over time, despite enhanced disease management.

Differences in sexual function impairment were also observed based on IBD type. Ghazi et al. [55] reported that women with Crohn’s disease (CD) exhibit higher rates of dyspareunia, reduced sexual desire, and difficulty achieving orgasm compared to those with ulcerative colitis (UC), likely due to perianal complications, chronic pain, and a greater need for surgical interventions.

Penetrating forms or forms involving perianal disease are often associated with a more pronounced impact on sexual health, with challenges related to pain, negative body perception, and limitations in intimate relationships [61].

On the other hand, Pires et al. [60] found that in patients with UC, sexual quality of life is primarily affected by disease activity and intestinal symptoms (diarrhea, urgency to defecate) rather than by permanent anatomical damage.

Disease activity is another key factor affecting sexual health. Nisihara et al. [59] reported that up to 80% of women with active IBD experience sexual dysfunction, compared to 45% of those in remission. Similarly, Ghazi et al. [55] found that more severe disease states were associated with a drastic decline in sexual interest and satisfaction, further confirming the link between inflammatory activity and sexual quality of life.

These findings are further supported by a study by Beattie [73], which demonstrated that patients with more active IBD are more likely to report a negative impact of their illness on sexual health.

Consequently, patients in the active phase of the disease exhibit significantly reduced levels of sexual function compared to those in remission, confirming the pivotal role of disease activity in the impairment of sexual health [21,74].

A study conducted by Ona et al. [75] demonstrated that vulvo-vaginal symptoms and pelvic pain, which are frequently observed in women with active IBD, are strongly correlated with compromised sexual health. These symptoms can affect not only sexual desire, but also the capacity to engage in fulfilling intimate experiences [75].

Chronic fatigue, as highlighted by a review conducted by McGing [76], is among the most prevalent symptoms of IBD, affecting up to 86% of patients. Numerous studies have reported that the impact of fatigue may even surpass that of the primary symptoms of the disease [76]. This state of persistent fatigue significantly affects sexual life, diminishing motivation and the desire to engage in intimate activities [77].

Surgical interventions, particularly the creation of ostomies, represents an additional vulnerability factor. Ghazi et al. [55] highlighted that ostomy patients encounter unique challenges related to the management of external appliances during sexual intercourse, with concerns about odor, gas, and leakage exacerbating discomfort. A phenomenological study conducted by Vural [78] further emphasized that women with bowel ostomies exhibit a significant decrease in sexual desire. Participants reported intentionally avoiding sexual intercourse and, in many cases, refraining from sharing a bed with their partners, revealing the profound and multifactorial impact of this condition on emotional and physical well-being [78].

Additionally, psychological factors such as anxiety and depression significantly affect individuals with IBD and are highly prevalent among these patients [79]. According to the literature, the prevalence of depressive disorders in IBD patients ranges from 21% to 25%, whereas anxiety disorders are observed in 19.1% to 35% of cases [80]. Zhang et al. and Ghazi et al. [55,63] emphasized that these conditions are critical predictors of impaired sexual health, aligning with the strong association between mental health and chronic disease highlighted by Bel et al. [21]. Among these factors, depression emerges as the most significant determinant of sexual problems [81]. Women with depression are more likely to report reduced sexual thoughts and desires, as well as a decline in the frequency of intimate relationships [68,82]. Additionally, evidence has demonstrated a negative relationship between depression and key aspects of sexual health, including body image [83], confidence during intimacy, sexual satisfaction [84,85], and family functioning [42,86].

Anxiety further intensifies challenges in sexual health, beyond the impairment associated with depression. It engenders profound fears related to body image and intimate relationships, creating a sense of entrapment as patients endeavor to balance their illness while maintaining personal connections [42,87]. This results in significant challenges in sexual health, adversely affecting sexual satisfaction, body image, and self-awareness during intimacy [42,63].

These findings align with the study by Shifren et al. [88], who revealed that women with high levels of anxiety were 33–50% more likely to experience poor sexual health compared to non-anxious women.

A key finding of our study is the strong connection between perceptions of sexual health and body image among women with IBD. The study conducted by McDermott et al. [89] highlighted how dissatisfaction with one’s body, often exacerbated by steroid use and the physical consequences of the disease, negatively affects self-esteem and sexual satisfaction. This discomfort is consistent among patients with Crohn’s disease and those with ulcerative rectocolitis, underlining a cross-sectional impact independent of the specific type of disease [89].

The perception of being ’damaged’ or ’mutilated’ profoundly undermines self-esteem, generating feelings of inadequacy and shame. Patients experience socially embarrassing symptoms that can inhibit their sexual contact and satisfaction [21,22,54]. According to the findings of studies conducted by Muller et al. [90], 50.2% of participants reported that the disease negatively affected their sexual health, with a greater impact observed in patients who had undergone surgery compared to those who had not.

Despite these difficulties, some studies have highlighted the importance of supportive relationships. Pires et al. [60] demonstrate that empathetic partners who are open to dialogue can mitigate the negative impact of the disease, improving the quality of the relationship. However, lack of communication remains a significant barrier, with many patients unsure of how to broach the subject with their partners, exacerbating feelings of loneliness [37,91].

The qualitative study conducted by Fourie et al. [53] investigates the challenges women face in discussing issues related to their sexuality. Many women report difficulties in openly communicating about their sexual needs, whether with partners or health professionals. This sense of isolation is both interpersonal and systemic, as sexual health is frequently neglected during clinical visits [37,51,92]. Notably, only 14% of gastroenterologists routinely inquire about the sexual health of their patients [51,93]. Miki and Hohashi et al. [94] highlighted that numerous healthcare professionals are reluctant to address issues related to sexuality, citing reasons such as time constraints, inadequate training, and personal discomfort. Ghazi et al. [55] report that many patients experience disappointment when doctors/physicians avoid addressing the topic of sexual function.

Patients frequently encounter these challenges in isolation, which intensifies their feelings of loneliness and frustration. Consequently, a significant theme identified in studies is the insufficient attention given to sexual health during medical consultations [64,92]. Factors such as limited time, inadequate training, and personal discomfort contribute to this oversight [95,96]. Marin et al. [44] reported that 64% of women and nearly half of men expressed a desire for information regarding the impact of IBD on sexuality at the time of diagnosis. Sexual topics, as emphasized in the study by Rasmussen Edelbo et al. [97], are often considered taboo by both patients with IBD and healthcare professionals. According to the study, 86% of patients reported not discussing sexual issues with the medical staff. Among them, 38% wished for healthcare professionals to address the topic, 34% perceived it as a taboo, and 25% found it challenging to discuss [97]. These data underscore the necessity for health services that are more attuned to and focused on the sexual health of IBD patients. Indeed, Fourie et al. [53] emphasized that patients desire a more holistic approach, including regular discussions about sexual well-being. The lack of proper education and counselling, as noted by Ellul et al. [27], results in uncertainties and misinterpretations that further compromise the quality of the personal, social, and marital lives of patients.

Communication barriers, such as embarrassment or the belief that sexuality is a secondary issue in managing the disease, often prevent discussion of these issues [71]. To facilitate open dialogue, it is crucial to adopt an empathetic and non-invasive approach, employing open-ended questions that enable patients to express themselves freely without fear of judgment.

Employing, neutral and inclusive language serves to normalize the conversation, thereby facilitating patients’ ability to articulate any challenges they may encounter. Furthermore, establishing a private and non-judgmental environment is crucial for fostering honest and constructive dialogue [71,96].

Effective communication regarding sexual health in individuals with IBD necessitates the use of personalized communication tools, appropriate training for healthcare professionals, and a patient-centered approach that considers the specific needs of the patient [71,96,98].

As highlighted in a review by Manning [99], sexual communication is frequently neglected in studies of interpersonal communication. However, it is a fundamental component of health and well-being, with direct implications for the prevention, health education, and management of sexual dysfunction. A primary gap identified is the paucity of studies analyzing doctor–patient dialogue on these topics and the inadequate training of health professionals. As suggested by Manning, overcoming a heteronormative and medicalized communication model is essential for a more inclusive approach to sexual health [99]. A more adaptable and personalized communication style could enhance therapeutic relationships, fostering greater trust and improved adherence to treatment pathways [97].

Moreover, perceptions of sexual health in patients with IBD are significantly influenced by the cultural context, with notable differences in how the topic is addressed in clinical settings. Cultural norms, stigma, and religious beliefs can affect patients’ willingness to openly discuss sexual well-being with healthcare professionals. In some communities, sexuality is considered a taboo subject, and patients may feel ashamed or uncomfortable discussing these issues due to fear of judgment or misunderstanding [100,101]. Studies have demonstrated that cultural barriers can increase feelings of isolation among patients with IBD, particularly among ethnic and sexual minorities, thereby limiting access to appropriate care and the recognition of sexual health problems [102,103]. These differences highlight the importance of a culturally sensitive approach to patient–provider communication. In addition to facilitating discussions about sexual health, health professionals must be cognizant of the cultural and religious influences that shape patients’ experiences and expectations [104].

Sexual problems in individuals with IBD disrupt social relationships, reduce personal skills, and diminish productivity [81]. The stigma associated with IBD exacerbates psychological distress, adversely affecting sexual health by negatively influencing intimacy and sexual satisfaction [42,105]. While patients often experience embarrassment regarding their condition, they express a strong desire for sexual health to be openly addressed during discussions with healthcare professionals. This was highlighted in a phenomenological study by Simeone et al., which explored the role of stigma in shaping the lived experiences of patients with IBD [19].

Multidisciplinary interventions have emerged as promising solutions to address these challenges. Ellul et al. and Igerc et al. [27,56] demonstrated that educational programs and targeted therapies, such as pelvic floor rehabilitation, biofeedback, and muscle relaxation techniques, can alleviate pelvic pain and enhance the sexual quality of life.

Furthermore, standardized tools such as the Female Sexual Function Index (FSFI) and Sexual Quality of Life Questionnaire Female (SQoL-F) facilitate the early identification of problems, enabling clinicians to tailor interventions [106,107]. The FSFI is a reliable questionnaire for assessing sexual dysfunction in women with IBD owing to its robust psychometric properties and validation in different clinical settings [108,109]. It measures desire, arousal, lubrication, orgasm, satisfaction, and pain and has been validated in numerous populations, including those with chronic disease [107].

The FSFI does not cover IBD-specific aspects such as flares, ostomies, or surgical complications [107,109]. Consequently, some studies complement the FSFI with more specific tools, such as the SQoLQ, which is designed to assess the impact of the disease on sexual quality of life [110,111].

Unlike the FSFI, which focuses on sexual function, the SQoLQ provides a broader perspective of overall sexual well-being. It has been shown to be reliable, valid, and sensitive to changes over time, making it useful for monitoring the impact of the condition and the effectiveness of therapeutic interventions. Studies have demonstrated significant differences in scores between screening and treatment phases, confirming their sensitivity to clinical changes [112,113].

The SQoLQ is particularly useful for identifying the most relevant areas for intervention in patients with IBD, as it addresses specific aspects of their sexual health and links them to the overall quality of life [110].

It is imperative that these tools be incorporated into a comprehensive framework that acknowledges women’s lived experiences as an essential component of the assessment [44,73]. Merely quantifying sexual function is insufficient: it is vital to comprehend the significance that patients attribute to sexuality and its integration with their identity and overall well-being.

Transformations in clinical practice are indispensable. As suggested by Martin and Woodgate at al. [114], healthcare professionals must actively engage in addressing sexual well-being needs. This approach, combining emotional sensitivity and technical expertise, can increase patients’ self-esteem, improve sexual relationships, and restore a sense of control over the disease [115,116]

Finally, the approach to sexual health in IBD must be personalized, taking into account each patient’s age, sex, and medical history [57]. Despite sexual health often being neglected, it contributes significantly to the quality of life of women with IBD [73]. This review aimed to synthesize the fragmented evidence regarding the sexual health of women with IBD. This study differs from previous meta-analyses and reviews of sexual health on people with IBD because of its multidimensional approach. While many studies have focused predominantly on the medical and physiological aspects of sexual dysfunction, this analysis also integrates psychological, relational, and clinical communication factors, elements which have been neglected in previous research. Another innovative aspect of this work is the specific focus on women’s sexual health, which allows an in-depth examination of the unique challenges and perceptions faced by women with IBD, thereby transcending generalized analyses of the broader IBD population. The results of this review confirm the evidence reported in previous studies on the high level of impairment of sexual health in women with IBD and the role of the disease in altering sexual function and psychological well-being. However, this review distinguishes itself by emphasizing clinical communication, the experiences of women with IBD, and the necessity of integrating sexual health into the comprehensive management of the disease.

The need emerging for a holistic approach that acknowledges and values the unique experiences and challenges encountered by these patients is related to body image, symptom management, and intimate relationships.

By integrating comprehensive screening and treatment guidelines, healthcare professionals can not only address physical difficulties, but can also respond to the psychological and emotional implications that significantly impact this dimension of well-being [108,117]. An empathic and focused approach enables patients to feel heard and understood, improving their overall quality of life [73,118]. This approach facilitates the development of a more positive relationship with their bodies and allows them to manage their illness with greater serenity and control.

Targeted attention to these dimensions not only not only enhances the sense of dignity and personal worth among women with IBD but also assists them in regaining a balance in experiencing their sexuality in a fulfilling manner.

### 4.1. Limits of the Revision

This narrative review possesses methodological limitations that warrant consideration when interpreting the findings. Primarily, the narrative format of the review precludes a systematic analysis of the available data, rendering the results vulnerable to selection bias. Furthermore, the variability in study designs, measurement tools, and geographical contexts among the included studies may constrain the generalizability of the conclusions. A significant limitation is the predominant focus of the reviewed literature on Western populations, which may not adequately reflect the influence of cultural and geographical differences on sexual health in women with IBD. Cultural norms, stigma, and healthcare access vary considerably across different regions, potentially affecting the perception, reporting, and management of sexual health concerns in clinical settings. The paucity of studies from non-Western regions underscores a gap in the literature, thereby limiting the applicability of the findings to diverse patient populations. An additional limitation pertains to potential publication bias, which may skew the available literature on sexual health in women with IBD. There is a tendency to publish studies that emphasize significant associations or evident problems, while research with less alarming or non-statistically significant results may be underrepresented. This phenomenon could influence the overall perception of sexual health in women with IBD, providing a partial view of the issue and restricting a more balanced and comprehensive understanding of the topic. Another limitation is the scarcity of longitudinal studies exploring the long-term dynamics of sexual health in women with IBD. Most existing research relies on cross-sectional designs, which do not capture changes over time or the effects of disease progression and treatment modifications on sexual function. Finally, many studies do not incorporate targeted interventions or specific assessments of barriers to communication between patients and healthcare professionals, leaving a significant gap in the literature.

### 4.2. Implications for Clinical Practice

**Figure 3:**
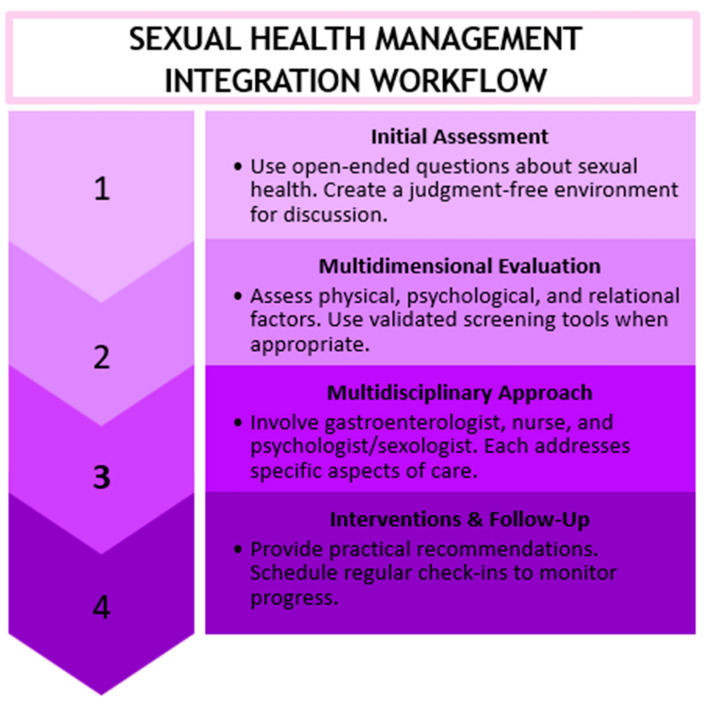
Workflow model for sexual health management.

Sexual health in women with IBD is frequently neglected during routine medical visits, despite its considerable impact on quality of life. Incorporating a structured assessment of sexual function into clinical practice can enhance the identification and management of sexual concerns. However, the absence of specific guidelines and practical tools is a barrier for healthcare professionals. Drawing on the existing literature, a sexual health management checklist and workflow model (Figure 3) have been proposed to aid healthcare providers in integrating sexual health as a fundamental component of patient care for women with IBD.

Sexual Health Management Checklist for Women with IBD:

General Medical History
  - Investigate changes in sexual function since the onset of the disease;
  - Consider relevant physical symptoms (pain, fatigue, and bowel problems during intercourse).
Psychological and Relational Well-Being
  - Explore the impact of the disease on self-perception and intimacy;
  - Assess potential difficulties in communicating about sexuality with a partner.
Clinical and Therapeutic Factors
  - Evaluate the effects of current medications (immunosuppressants, corticosteroids, and antidepressants) on sexual function;
  - Assess the impact of surgical procedures (e.g., ostomy) on sexual quality of life.
Support and Management
  - Promote an open and non-judgmental environment for discussing sexual health concerns;
  - Use clear and sensitive language to normalize discussions about sexual health;
  - Provide information about symptom management strategies (pelvic floor physiotherapy, relaxation techniques, and mindfulness);
  - Consider involving specialists (sexologists, psychologists, and pelvic rehabilitation therapists).

### 4.3. Implications for Future Research

To enhance the understanding and management of sexual health in women with IBD, further research is required to address these identified limitations. Longitudinal studies with representative samples and multicenter designs can provide a more comprehensive view of patients’ experiences.

Future studies should incorporate longitudinal data collection to elucidate how sexual health evolves over time in women with IBD, considering factors such as disease activity, treatment modifications, and psychological adaptation. Unlike cross-sectional studies, which provide only a snapshot at a single point in time, longitudinal research can investigate dynamic changes in sexual function and identify patterns of improvement or deterioration related to disease progression, remission phases, and therapeutic interventions.

Additionally, longitudinal data would enable researchers to assess the long-term effectiveness of personalized interventions and analyze whether structured communication programs, psychological support, or pelvic floor rehabilitation produce sustainable benefits for sexual health. By tracking patients across different stages of the disease and treatment, these studies could contribute to the development of evidence-based guidelines to improve both clinical practice and patient quality of life.

Furthermore, it is essential to develop targeted interventions and standardized tools to address the communication barriers between patients and healthcare professionals.

Future research should investigate cultural differences in the perception of sexual health and evaluate the effectiveness of multidisciplinary approaches and educational interventions. Finally, it is crucial to incorporate patients’ perspectives in the design of studies to ensure that the results are relevant and applicable to clinical practice. Such initiatives could help to fill existing gaps and promote a holistic, patient-centered approach to the management of IBD.

## 5. Conclusions

This narrative review highlights the profound impact of IBD on women’s sexual health, influencing not only physical well-being but also psychological and relational equilibrium. Despite the significance of these issues, sexual health remains a marginal aspect of IBD clinical management, frequently neglected by healthcare professionals and rarely discussed with patients.

To enhance the quality of life for women with IBD, it is essential to adopt a holistic and personalized approach that incorporates sexual health as a fundamental component of clinical care. This necessitates increased awareness among healthcare professionals, the development of multidisciplinary support strategies, and targeted interventions that consider the complexity of patients’ experiences. Only through a structural transformation in the approach to care can effective support be ensured, thereby improving the sexual health of women with IBD.

## Figures and Tables

**Table 1:** QuADS tool (Quality Assessment with Diverse Studies).

Study	Theoretical or Conceptual Underpinning	Research Aim Statement	Research Setting and Target Population	Study Design Appropriateness	Sampling Appropriateness	Rationale for Data Collection Tools	Data Collection Tool Format and Content	Description of Data Collection Procedure	Recruitment Data Provided	Justification for Analytic Method	Appropriateness of Data Analysis	Consideration of Research Stakeholders	Strengths and Limitations Critically Discussed	Total Score (Max 39)	Percentage Total Score
Boyd [51]	3	2	2	2	1	1	1	0	0	0	0	2	1	27	41%
Domislovic [52]	3	3	3	3	2	3	3	2	2	1	3	3	3	35	89%
Ellul [27]	2	3	2	3	3	3	3	2	3	2	3	3	3	35	89%
Fourie [53]	3	3	3	3	2	3	3	2	2	3	3	3	3	34	87%
Fretz [54]	3	3	3	3	2	3	3	2	2	2	3	3	3	34	87%
Ghazi [55]	2	2	3	2	2	2	2	1	1	1	2	2	2	24	61%
Igerc [56]	3	3	3	2	2	2	2	2	2	1	2	2	1	27	69%
Kanar [57]	3	3	3	3	2	3	3	2	2	2	3	3	3	35	89%
Knoweles [42]	3	3	3	3	2	3	3	2	2	1	3	3	3	34	87%
McIntosh [58]	3	3	3	3	2	3	3	2	2	2	2	2	2	32	82%
Nisihara [59]	3	3	3	3	3	2	3	2	2	2	2	3	2	33	84%
Pires [60]	3	3	3	3	2	3	2	2	2	2	3	3	3	34	87%
Smith [61]	3	3	3	3	2	3	2	2	2	3	3	3	3	35	89%
White [62]	2	2	2	2	1	2	2	1	1	1	2	2	1	21	53%
Zhang [45]	3	3	3	3	3	3	2	2	3	2	3	3	2	35	89%

## Data Availability

No new data were created or analyzed in this study. Data sharing is not applicable to this article.

## References

[1] Kaser A., Zeissig S., Blumberg R.S. (2010). Inflammatory Bowel Disease. Annu. Rev. Immunol..

[2] Rubin D.C., Shaker A., Levin M.S. (2012). Chronic Intestinal Inflammation: Inflammatory Bowel Disease and Colitis-Associated Colon Cancer. Front. Immunol..

[3] Fakhoury M., Al-Salami H., Negrulj R., Mooranian A. (2014). Inflammatory Bowel Disease: Clinical Aspects and Treatments. J. Inflamm. Res..

[4] Jairath V., Feagan B.G. (2020). Global Burden of Inflammatory Bowel Disease. Lancet Gastroenterol. Hepatol..

[5] Kilic Y., Kamal S., Jaffar F., Sriranganathan D., Quraishi M.N., Segal J.P. (2024). Prevalence of Extraintestinal Manifestations in Inflammatory Bowel Disease: A Systematic Review and Meta-Analysis. Inflamm. Bowel Dis..

[6] Urlep D., Mamula P., Baldassano R. (2005). Extraintestinal Manifestations of Inflammatory Bowel Disease. Minerva Gastroenterol. Dietol..

[7] Jones J.L., Nguyen G.C., Benchimol E.I., Bernstein C.N., Bitton A., Kaplan G.G., Murthy S.K., Lee K., Cooke-Lauder J., Otley A.R. (2019). The Impact of Inflammatory Bowel Disease in Canada 2018: Quality of Life. J. Can. Assoc. Gastroenterol..

[8] Jordan C., Sin J., Fear N.T., Chalder T. (2016). A Systematic Review of the Psychological Correlates of Adjustment Outcomes in Adults with Inflammatory Bowel Disease. Clin. Psychol. Rev..

[9] Kemp K. (2012). Understanding the Health and Social Care Needs of People Living with IBD: A Meta-Synthesis of the Evidence. World J. Gastroenterol..

[10] Barberio B., Zamani M., Black C.J., Savarino E.V., Ford A.C. (2021). Prevalence of Symptoms of Anxiety and Depression in Patients with Inflammatory Bowel Disease: A Systematic Review and Meta-Analysis. Lancet Gastroenterol. Hepatol..

[11] Neuendorf R., Harding A., Stello N., Hanes D., Wahbeh H. (2016). Depression and Anxiety in Patients with Inflammatory Bowel Disease: A Systematic Review. J. Psychosom. Res..

[12] Opheim R., Fagermoen M.S., Bernklev T., Jelsness-Jorgensen L.-P., Moum B. (2014). Fatigue Interference with Daily Living among Patients with Inflammatory Bowel Disease. Qual. Life Res..

[13] Lönnfors S., Vermeire S., Greco M., Hommes D., Bell C., Avedano L. (2014). IBD and Health-Related Quality of Life—Discovering the True Impact. J. Crohns Colitis.

[14] Rogler G., Singh A., Kavanaugh A., Rubin D.T. (2021). Extraintestinal Manifestations of Inflammatory Bowel Disease: Current Concepts, Treatment, and Implications for Disease Management. Gastroenterology.

[15] Salem J., Ghandour F., Halabi M.T., Douaihy T., Matta J., Bedran K., Farhat S. (2022). P367 Quality of Life in Patients with Inflammatory Bowel Disease. J. Crohns Colitis.

[16] Clearfield H.R. (2008). How Does IBD Affect Quality of Life?. Inflamm. Bowel Dis..

[17] Colonnello V., Agostini A. (2020). Disease Course, Stress, Attachment, and Mentalization in Patients with Inflammatory Bowel Disease. Med. Hypotheses.

[18] Larussa T., Flauti D., Abenavoli L., Boccuto L., Suraci E., Marasco R., Imeneo M., Luzza F. (2020). The Reality of Patient-Reported Outcomes of Health-Related Quality of Life in an Italian Cohort of Patients with Inflammatory Bowel Disease: Results from a Cross-Sectional Study. J. Clin. Med..

[19] Simeone S., Mercuri C., Cosco C., Bosco V., Pagliuso C., Doldo P. (2023). Enacted Stigma in Inflammatory Bowel Disease: An Italian Phenomenological Study. Healthcare.

[20] Vaughan H., Jolliffe T. (2023). ‘Why It’s Important to Talk about Our Toilet Needs in the Workplace’—Using Maslow’s Needs Theory to Shine a Light on Workers Living with IBD in the Workplace. Qual. Rep..

[21] Bel L.G.J., Vollebregt A.M., Van Der Meulen-de Jong A.E., Fidder H.H., Ten Hove W.R., Vliet-Vlieland C.W., Ter Kuile M.M., De Groot H.E., Both S. (2015). Sexual Dysfunctions in Men and Women with Inflammatory Bowel Disease: The Influence of IBD-Related Clinical Factors and Depression on Sexual Function. J. Sex. Med..

[22] Lindenthal D., Kranzeder A., Hirning C., Von Wietersheim J., Klaus J. (2023). P073 Let′s Talk about Sex: An Unicentric Cross-Sectional Study on Sexual Dysfunction, Sexual Satisfaction and Body Schema Disorders in Inflammatory Bowel Disease. J. Crohns Colitis.

[23] Flynn K.E., Lin L., Bruner D.W., Cyranowski J.M., Hahn E.A., Jeffery D.D., Reese J.B., Reeve B.B., Shelby R.A., Weinfurt K.P. (2016). Sexual Satisfaction and the Importance of Sexual Health to Quality of Life Throughout the Life Course of U.S. Adults. J. Sex. Med..

[24] World Health Organization (2006). Defining Sexual Health: Report of a Technical Consultation on Sexual Health, 28–31 January 2002, Geneva.

[25] Connelly T.M., Berg A.S., Harris L., Brinton D., Deiling S., Koltun W.A. (2015). Genetic Determinants Associated With Early Age of Diagnosis of IBD. Dis. Colon Rectum.

[26] Cushman G., Stolz M.G., Shih S., Listernick Z., Talmadge C., Gold B.D., Reed B. (2021). Age, Disease Symptoms, and Depression Are Associated With Body Image Dissatisfaction in Newly Diagnosed Pediatric Inflammatory Bowel Disease. J. Pediatr. Gastroenterol. Nutr..

[27] Ellul P., Zammit S.C., Katsanos K.H., Cesarini M., Allocca M., Danese S., Karatzas P., Moreno S.C., Kopylov U., Fiorino G. (2016). Perception of Reproductive Health in Women with Inflammatory Bowel Disease. J. Crohns Colitis.

[28] Mancheron A., Dumas A., Martinez-Vinson C., Bourmaud A. (2024). Impact of Inflammatory Bowel Diseases on the Intimate Lives of Youths: Creating a Brief Screening Questionnaire. J. Pediatr. Gastroenterol. Nutr..

[29] Picciarelli Z., Stransky O.M., Leech M.M., Michel H.K., Schwartz M., Kim S.C., Gray W.M., Kazmerski T.M. (2022). Exploring Reproductive Health Decision Experiences and Preferences of Women With Pediatric-Onset Inflammatory Bowel Diseases. Crohns Colitis 360.

[30] Shannahan S.E., Erlich J.M., Peppercorn M.A. (2019). Insights into the Treatment of Inflammatory Bowel Disease in Pregnancy. Ther. Adv. Gastroenterol..

[31] Kotkowicz-Szczur M., Szymańska E., Kisielewski R., Kierkuś J. (2023). Sexual Functions in Individuals with Inflammatorybowel Diseases. Gastroenterol. Rev..

[32] Lix L.M., Graff L.A., Walker J.R., Clara I., Rawsthorne P., Rogala L., Miller N., Ediger J., Pretorius T., Bernstein C.N. (2008). Longitudinal Study of Quality of Life and Psychological Functioning for Active, Fluctuating, and Inactive Disease Patterns in Inflammatory Bowel Disease. Inflamm. Bowel Dis..

[33] Pizzi L.T., Weston C.M., Goldfarb N.I., Moretti D., Cobb N., Howell J.B., Infantolino A., DiMarino A.J., Cohen S. (2006). Impact of Chronic Conditions on Quality of Life in Patients with Inflammatory Bowel Disease. Inflamm. Bowel Dis..

[34] Timmer A., Bauer A., Kemptner D., Fürst A., Rogler G. (2007). Determinants of Male Sexual Function in Inflammatory Bowel Disease: A Survey-Based Cross-Sectional Analysis in 280 Men. Inflamm. Bowel Dis..

[35] Timmer A., Kemptner D., Bauer A., Takses A., Ott C., Fürst A. (2008). Determinants of Female Sexual Function in Inflammatory Bowel Disease: A Survey Based Cross-Sectional Analysis. BMC Gastroenterol..

[36] Mantzouranis G., Fafliora E., Glanztounis G., Christodoulou D.K., Katsanos K.H. (2015). Inflammatory Bowel Disease and Sexual Function in Male and Female Patients: An Update on Evidence in the Past Ten Years. J. Crohns Colitis.

[37] Trachter A.B., Rogers A.I., Leiblum S.R. (2002). Inflammatory Bowel Disease in Women: Impact on Relationship and Sexual Health. Inflamm. Bowel Dis..

[38] Black P.K. (2004). Psychological, Sexual and Cultural Issues for Patients with a Stoma. Br. J. Nurs..

[39] Junkin J., Beitz J.M. (2005). Sexuality and the Person With a Stoma: Implications for Comprehensive WOC Nursing Practice. J. Wound. Ostomy Cont. Nurs..

[40] Kralik D., Koch T., Telford K. (2001). Constructions of Sexuality for Midlife Women Living with Chronic Illness. J. Adv. Nurs..

[41] Li C.-C., Rew L. (2010). A Feminist Perspective on Sexuality and Body Image in Females With Colorectal Cancer: An Integrative Review. J. Wound. Ostomy Cont. Nurs..

[42] Knowles S.R., Gass C., Macrae F. (2013). Illness Perceptions in IBD Influence Psychological Status, Sexual Health and Satisfaction, Body Image and Relational Functioning: A Preliminary Exploration Using Structural Equation Modeling. J. Crohns Colitis.

[43] Nkansah M., Alzweri L. (2023). (315) The Association of Sexual Dysfunction with Race in Men and Women: A Comparative Review. J. Sex. Med..

[44] Marín L., Mañosa M., Garcia-Planella E., Gordillo J., Zabana Y., Cabré E., Domènech E. (2013). Sexual Function and Patients’ Perceptions in Inflammatory Bowel Disease: A Case–Control Survey. J. Gastroenterol..

[45] Zhang J., Nie J., Zou M., Zeng Q., Feng Y., Luo Z., Gan H. (2022). Prevalence and Associated Factors of Sexual Dysfunction in Patients With Inflammatory Bowel Disease. Front. Endocrinol..

[46] Sarkar S., Bhatia G. (2021). Writing and Appraising Narrative Reviews. J. Clin. Sci. Res..

[47] Grant M.J., Booth A. (2009). A Typology of Reviews: An Analysis of 14 Review Types and Associated Methodologies. Health Inf. Libr. J..

[48] Page M.J., McKenzie J.E., Bossuyt P.M., Boutron I., Hoffmann T.C., Mulrow C.D., Shamseer L., Tetzlaff J.M., Akl E.A., Brennan S.E. (2021). The PRISMA 2020 Statement: An Updated Guideline for Reporting Systematic Reviews. BMJ.

[49] Al-Shaari H., Heales C.J. (2023). A Systematic Review of Repeatability and Reproducibility Studies of Diffusion Tensor Imaging of Cervical Spinal Cord. Br. J. Radiol..

[50] Harrison R., Jones B., Gardner P., Lawton R. (2021). Quality Assessment with Diverse Studies (QuADS): An Appraisal Tool for Methodological and Reporting Quality in Systematic Reviews of Mixed- or Multi-Method Studies. BMC Health Serv. Res..

[51] Boyd T., De Silva P.S., Friedman S. (2022). Sexual Dysfunction in Female Patients with Inflammatory Bowel Disease: An Overview. Clin. Exp. Gastroenterol..

[52] Domislovic V., Brinar M., Cukovic-Cavka S., Turk N., Mikolasevic I., Krznaric Z. (2021). Prevalence, Predictors and Age-related Sexual and Erectile Dysfunction in Patients with Inflammatory Bowel Disease: A Tertiary Centre Experience. Int. J. Clin. Pract..

[53] Fourie S., Jackson D., Aveyard H. (2018). Living with Inflammatory Bowel Disease: A Review of Qualitative Research Studies. Int. J. Nurs. Stud..

[54] Fretz K.M., Hunker K.E., Tripp D.A. (2024). The Impact of Inflammatory Bowel Disease on Intimacy: A Multimethod Examination of Patients’ Sexual Lives and Associated Healthcare Experiences. Inflamm. Bowel Dis..

[55] Ghazi L.J., Patil S.A., Cross R.K. (2015). Sexual Dysfunction in Inflammatory Bowel Disease. Inflamm. Bowel Dis..

[56] Igerc I., Schrems B. (2023). Sexual Well-being Needs of Patients with Chronic Illness Expressed in Health Care: A Scoping Review. J. Clin. Nurs..

[57] Kanar O., Berry A.C., Nakshabendi R., Lee A.J., Aldridge P., Myers T., Eid E. (2017). Effects of Immunomodulators and Biologic Agents on Sexual Health in Patients With Inflammatory Bowel Disease. Ochsner J..

[58] McIntosh S., Pardoe H., Brown K. (2013). Effect of Colorectal Cancer Surgery on Female Sexual Function: A Prospective Cohort Study. Gastrointest. Nurs..

[59] Nisihara R., Schulz A.F.C., Conrado B.A., Ramos Júnior O., Sobreiro B., Skare T. (2020). Sexual Dysfunction in Patients with Inflammatory Bowel Disease. Sex. Disabil..

[60] Pires F., Martins D., Ministro P. (2022). A Survey on the Impact of IBD in Sexual Health: Into Intimacy. Medicine.

[61] Smith J.A., Spiers J., Simpson P., Nicholls A.R. (2017). The Psychological Challenges of Living with an Ileostomy: An Interpretative Phenomenological Analysis. Health Psychol..

[62] White C. (2013). Sexual Health Following Stoma Surgery. Gastrointest. Nurs..

[63] Zhang J., Wei S., Zeng Q., Wu X., Gan H. (2021). Prevalence and Risk Factors of Sexual Dysfunction in Patients with Inflammatory Bowel Disease: Systematic Review and Meta-Analysis. Int. J. Colorectal Dis..

[64] Leenhardt R., Rivière P., Papazian P., Nion-Larmurier I., Girard G., Laharie D., Marteau P. (2019). Sexual Health and Fertility for Individuals with Inflammatory Bowel Disease. World J. Gastroenterol..

[65] Rivière P., Zallot C., Desobry P., Sabaté J.M., Vergniol J., Zerbib F., Peyrin-Biroulet L., Laharie D., Poullenot F. (2017). Frequency of and Factors Associated With Sexual Dysfunction in Patients With Inflammatory Bowel Disease. J. Crohns Colitis.

[66] Verschuren J.E.A., Enzlin P., Dijkstra P.U., Geertzen J.H.B., Dekker R. (2010). Chronic Disease and Sexuality: A Generic Conceptual Framework. J. Sex Res..

[67] Basson R., Rees P., Wang R., Montejo A.L., Incrocci L. (2010). Sexual Function in Chronic Illness. J. Sex. Med..

[68] Peixoto M.M. (2024). Female Sexual Desire and Trait-Affect: The Mediator Role of Depressed Mood. Sex. Relatsh. Ther..

[69] Valera-Ribera C., Robustillo-Villarino M., Flores-Fernández E., Andújar-Brazal P., Vázquez-Gómez I., Ybañez Garcia A., Martínez-Ferrer À., Valls-Pascual E., Alegre-Sancho J.J. (2022). OP0139 IMPACT OF CHRONIC JOINT DISEASES ON THE SEXUAL SPHERE WITH REGARDS TO A HEALTHY POPULATION: A MULTICENTER STUDY. Ann. Rheum. Dis..

[70] Lynch B., Connor L. (2022). Effects of Chronic Neurological Conditions on Sexual Activity Participation, Satisfaction, and Quality of Life. Am. J. Occup. Ther..

[71] Ma S., Knapp P., Galdas P. (2024). ‘My Sexual Desires, Everything, My Normal Life Just Stops’; a Qualitative Study of Male Sexual Health in Inflammatory Bowel Disease. J. Clin. Nurs..

[72] Shmidt E., Suárez-Fariñas M., Mallette M., Moniz H., Bright R., Shah S.A., Merrick M., Shapiro J., Xu F., Sands B. (2019). A Longitudinal Study of Sexual Function in Women With Newly Diagnosed Inflammatory Bowel Disease. Inflamm. Bowel Dis..

[73] Beattie W., Elford A., Segal J., Kaushik V., Downie A., Mitchell J., Al-Ani A., Prentice R., Christensen B. (2024). P1006 Assessing Sexual Health Care Needs in Patients with Inflammatory Bowel Disease. J. Crohns Colitis.

[74] Mules T.C., Swaminathan A., Hirschfeld E., Borichevsky G.M., Frampton C.M., Day A.S., Gearry R.B. (2023). The Impact of Disease Activity on Sexual and Erectile Dysfunction in Patients With Inflammatory Bowel Disease. Inflamm. Bowel Dis..

[75] Ona S., James K., Ananthakrishnan A.N., Long M.D., Martin C., Chen W., Mitchell C.M. (2020). Association Between Vulvovaginal Discomfort and Activity of Inflammatory Bowel Diseases. Clin. Gastroenterol. Hepatol..

[76] McGing J.J., Radford S.J., Francis S.T., Serres S., Greenhaff P.L., Moran G.W. (2021). Review Article: The Aetiology of Fatigue in Inflammatory Bowel Disease and Potential Therapeutic Management Strategies. Aliment. Pharmacol. Ther..

[77] Wakai S., Tanaka M., Takai M., Sakagami K., Hiroaki I. (2024). N13 Sexual Satisfaction and Its Associated Factors among Patients with Inflammatory Bowel Disease in Japan. J. Crohns Colitis.

[78] Vural F., Harputlu D., Karayurt O., Suler G., Edeer A.D., Ucer C., Onay D.C. (2016). The Impact of an Ostomy on the Sexual Lives of Persons With Stomas: A Phenomenological Study. J. Wound. Ostomy Cont. Nurs..

[79] Fracas E., Costantino A., Vecchi M., Buoli M. (2023). Depressive and Anxiety Disorders in Patients with Inflammatory Bowel Diseases: Are There Any Gender Differences?. Int. J. Environ. Res. Public Health.

[80] Bisgaard T.H., Allin K.H., Keefer L., Ananthakrishnan A.N., Jess T. (2022). Depression and Anxiety in Inflammatory Bowel Disease: Epidemiology, Mechanisms and Treatment. Nat. Rev. Gastroenterol. Hepatol..

[81] Ates Bulut E., Toruner M., Department of Geriatric Medicine, Dokuz Eylul University School of Medicine, Izmir, Turkey, Department of Gastroenterology, Ankara University School of Medicine, Ankara, Turkey (2018). The Influence of Disease Type and Activity to Sexual Life and Health Quality in Inflammatory Bowel Disease. Turk. J. Gastroenterol..

[82] Fabre L.F., Smith L.C. (2012). The Effect of Major Depression on Sexual Function in Women. J. Sex. Med..

[83] Kogure G.S., Ribeiro V.B., Lopes I.P., Furtado C.L.M., Kodato S., Silva De Sá M.F., Ferriani R.A., Lara L.A.D.S., Maria Dos Reis R. (2019). Body Image and Its Relationships with Sexual Functioning, Anxiety, and Depression in Women with Polycystic Ovary Syndrome. J. Affect. Disord..

[84] Carcedo R.J., Fernández-Rouco N., Fernández-Fuertes A.A., Martínez-Álvarez J.L. (2020). Association between Sexual Satisfaction and Depression and Anxiety in Adolescents and Young Adults. Int. J. Environ. Res. Public Health.

[85] Kim J.S., Kang S. (2015). A Study on Body Image, Sexual Quality of Life, Depression, and Quality of Life in Middle-Aged Adults. Asian Nurs. Res..

[86] Çömez İkican T., Coşansu G., Erdoğan G., Küçük L., Özel Bilim İ. (2020). The Relationship of Marital Adjustment and Sexual Satisfaction with Depressive Symptoms in Women. Sex. Disabil..

[87] Barnes E.L., Kochar B., Long M.D., Kappelman M.D., Martin C.F., Korzenik J.R., Crockett S.D. (2017). Modifiable Risk Factors for Hospital Readmission Among Patients with Inflammatory Bowel Disease in a Nationwide Database. Inflamm. Bowel Dis..

[88] Shifren J.L., Monz B.U., Russo P.A., Segreti A., Johannes C.B. (2008). Sexual Problems and Distress in United States Women: Prevalence and Correlates. Obstet. Gynecol..

[89] McDermott E., Mullen G., Moloney J., Keegan D., Byrne K., Doherty G.A., Cullen G., Malone K., Mulcahy H.E. (2015). Body Image Dissatisfaction: Clinical Features, and Psychosocial Disability in Inflammatory Bowel Disease. Inflamm. Bowel Dis..

[90] Muller K.R., Prosser R., Bampton P., Mountifield R., Andrews J.M. (2010). Female Gender and Surgery Impair Relationships, Body Image, and Sexuality in Inflammatory Bowel Disease. Inflamm. Bowel Dis..

[91] Tough H., Brinkhof M.W.G., Siegrist J., Fekete C. (2018). The Impact of Loneliness and Relationship Quality on Life Satisfaction: A Longitudinal Dyadic Analysis in Persons with Physical Disabilities and Their Partners. J. Psychosom. Res..

[92] Sanders J.N., Gawron L.M., Friedman S. (2016). Sexual Satisfaction and Inflammatory Bowel Diseases: An Interdisciplinary Clinical Challenge. Am. J. Obstet. Gynecol..

[93] Christensen B. (2014). Inflammatory Bowel Disease and Sexual Dysfunction. Gastroenterol. Hepatol..

[94] Miki Y., Hohashi N. (2018). Actual Conditions of Sexuality and Sexual Perceptions among IBD Patients in Japan. J. Med. Care Res. Rev..

[95] Elford A.T., Beattie W., Downie A., Kaushik V., Mitchell J., Prentice R., Al-Ani A.H., Segal J., Christensen B. (2024). Sexual Dysfunction Is Prevalent in IBD but Underserved: A Need to Expand Specialised IBD Care. Frontline Gastroenterol..

[96] Nozawa M., Wakai S., Fourie S., Kawakami A., Tanaka M. (2024). N19 Nurses’ Difficulties in Supporting the Sexual Well-Being of Patients with Inflammatory Bowel Disease. J. Crohns Colitis.

[97] Rasmussen Edelbo R., Faurskov Møller L., Bager P. (2024). N20 Sexual Health in IBD Can Be Brought up with Help from Tools. J. Crohns Colitis.

[98] Borum M.L., Igiehon E., Shafa S. (2010). Physicians May Inadequately Address Sexuality in Women with Inflammatory Bowel Disease. Inflamm. Bowel Dis..

[99] Manning J. (2021). Communication Studies about Sex: Implications for Relationships, Health, Culture, and Identity. A Review. El Prof. Inf..

[100] Fourie S., Czuber-Dochan W., Norton C. (2023). N20 The Added Shame of IBD: Experiences of People from Various Ethnic Backgrounds Discussing Sexual Wellbeing in Clinical Settings. J. Crohns Colitis.

[101] Helman C. (2007). Culture, Health and Illness.

[102] Dhar C.P., Kaflay D., Dowshen N., Miller V.A., Ginsburg K.R., Barg F.K., Yun K. (2017). Attitudes and Beliefs Pertaining to Sexual and Reproductive Health Among Unmarried, Female Bhutanese Refugee Youth in Philadelphia. J. Adolesc. Health.

[103] Nyblade L., Stockton M., Nyato D., Wamoyi J. (2017). Perceived, Anticipated and Experienced Stigma: Exploring Manifestations and Implications for Young People’s Sexual and Reproductive Health and Access to Care in North-Western Tanzania. Cult. Health Sex..

[104] Matusitz J., Spear J. (2014). Effective Doctor–Patient Communication: An Updated Examination. Soc. Work Public Health.

[105] Drossman D.A., Patrick D.L., Mitchell C.M., Zagami E.A., Appelbaum M.I. (1989). Health-Related Quality of Life in Inflammatory Bowel Disease: Functional Status and Patient Worries and Concerns. Dig. Dis. Sci..

[106] Isidori A.M., Pozza C., Esposito K., Giugliano D., Morano S., Vignozzi L., Corona G., Lenzi A., Jannini E.A. (2010). Original Research—Outcomes Assessment: Development and Validation of a 6-Item Version of the Female Sexual Function Index (FSFI) as a Diagnostic Tool for Female Sexual Dysfunction. J. Sex. Med..

[107] Rosen C., Brown J., Heiman S., Leiblum C., Meston R., Shabsigh D., Ferguson R., D’Agostino R. (2000). The Female Sexual Function Index (FSFI): A Multidimensional Self-Report Instrument for the Assessment of Female Sexual Function. J. Sex Marital Ther..

[108] De Silva P.S., O’Toole A., Marc L.G., Ulysse C.A., Testa M.A., Julsgaard M., Ngyuen D., Ananthakrishnan A., Laursen T., Højgaard A. (2018). Development of a Sexual Dysfunction Scale for Women With Inflammatory Bowel Disease. Inflamm. Bowel Dis..

[109] Neijenhuijs K.I., Hooghiemstra N., Holtmaat K., Aaronson N.K., Groenvold M., Holzner B., Terwee C.B., Cuijpers P., Verdonck-de Leeuw I.M. (2019). The Female Sexual Function Index (FSFI)—A Systematic Review of Measurement Properties. J. Sex. Med..

[110] Roseira J., Magro F., Fernandes S., Simões C., Portela F., Vieira A.I., Patita M., Leal C., Lago P., Caldeira P. (2020). Sexual Quality of Life in Inflammatory Bowel Disease: A Multicenter, National-Level Study. Inflamm. Bowel Dis..

[111] Woodward J.M.B., Hass S.L., Woodward P.J. (2002). Reliability and Validity of the Sexual Life Quality Questionnaire (SLQQ). Qual. Life Res..

[112] Mancheron A., Dumas A., Nion Larmurier I., Landman C., Peyrin Biroulet L., Caron B., Baudry C., Allez M., Serrero M., Yahioune D. (2024). Development and Validation of a Sexual Quality of Life Score for Youths With Inflammatory Bowel Disease. J. Crohns Colitis.

[113] Maasoumi R., Lamyian M., Montazeri A., Azin S.A., Aguilar-Vafaie M.E., Hajizadeh E. (2013). The Sexual Quality of Life-Female (SQOL-F) Questionnaire: Translation and Psychometric Properties of the Iranian Version. Reprod. Health.

[114] Martin K.M., Woodgate R.L. (2020). Concept Analysis: The Holistic Nature of Sexual Well-Being. Sex. Relatsh. Ther..

[115] Kamp K.J., West P., Holmstrom A., Luo Z., Wyatt G., Given B. (2019). Systematic Review of Social Support on Psychological Symptoms and Self-Management Behaviors Among Adults With Inflammatory Bowel Disease. J. Nurs. Scholarsh..

[116] Sheehan J.L., Greene-Higgs L., Swanson L., Higgins P.D.R., Krein S.L., Waljee A.K., Saini S.D., Berinstein J.A., Mellinger J.L., Piette J.D. (2023). Self-Efficacy and the Impact of Inflammatory Bowel Disease on Patients’ Daily Lives. Clin. Transl. Gastroenterol..

[117] O’Toole A., De Silva P.S., Marc L.G., Ulysse C.A., Testa M.A., Ting A., Moss A., Korzenik J., Friedman S. (2018). Sexual Dysfunction in Men With Inflammatory Bowel Disease: A New IBD-Specific Scale. Inflamm. Bowel Dis..

[118] Pugliese D., Parisio L., Schepis T., Privitera G., Calvez V., Gasbarrini A., Armuzzi A. (2022). Patient-Reported Outcomes for the Assessment of Sexual Health AmongPatients Affected by Inflammatory Bowel Disease. Rev. Recent Clin. Trials.

